# Enhancing Long-Term Memory in Carbon-Nanotube-Based Optoelectronic Synaptic Devices for Neuromorphic Computing

**DOI:** 10.3390/nano14181501

**Published:** 2024-09-16

**Authors:** Seung Hun Lee, Hye Jin Lee, Dabin Jeon, Hee-Jin Kim, Sung-Nam Lee

**Affiliations:** 1 Department of IT Semiconductor Convergence Engineering, Tech University of Korea, Siheung 15073, Republic of Korea; 2 Department of Nano & Semiconductor Engineering, Tech University of Korea, Siheung 15073, Republic of Korea

**Keywords:** CNT, optoelectronic, synapse, neuromorphic

## Abstract

This study investigates the impact of spin-coating speed on the performance of carbon nanotube (CNT)-based optoelectronic synaptic devices, focusing on their long-term memory properties. CNT films fabricated at lower spin speeds exhibited a greater thickness and density compared to those at higher speeds. These denser films showed enhanced persistent photoconductivity, resulting in higher excitatory postsynaptic currents (EPSCs) and the prolonged retention of memory states after UV stimulation. Devices coated at a lower spin-coating speed of 2000 RPM maintained EPSCs above 70% for 3600 s, outperforming their higher-speed counterparts in long-term memory retention. Additionally, the study demonstrated that the learning efficiency improved with repeated UV stimulation, with fewer pulses needed to achieve the maximum EPSC in successive learning cycles. These findings highlight that optimizing spin-coating speeds can significantly enhance the performance of CNT-based synaptic devices, making them suitable for applications in neuromorphic computing and artificial neural networks requiring robust memory retention and efficient learning.

## 1. Introduction

Recent advances in artificial intelligence are challenging the limitations of classical computer architectures in processing large amounts of data [1,2,3,4,5,6]. As an alternative, there is a growing interest in neuromorphic systems, which offer an alternative by mimicking the ability of the human brain to perform computation and memory tasks simultaneously with high efficiency and low power consumption [1,2,3]. Among these neuromorphic systems, synaptic devices are gaining prominence for their ability to emulate both short-term and long-term learning behaviors [4,5]. Research in this area has explored various mechanisms, including memristors, optical materials, phase change materials, conductive bridges, and ferroelectrics, to develop effective two-terminal synaptic devices [7,8,9,10]. Recent studies have focused on devices that exhibit key synaptic plasticity phenomena such as excitatory postsynaptic currents (EPSCs), paired-pulse facilitation (PPF), spike-timing-dependent plasticity, and spike-rate-dependent plasticity [10,11,12]. Optoelectronic synapses represent an advanced approach within this field, utilizing light stimulation rather than electrical signals. This shift aims to reduce energy consumption while enhancing robustness and signal transmission. In these devices, light signals function similarly to the gate, source, and drain electrodes in traditional flash memories, enabling the emulation of synaptic functions with improved efficiency [13,14,15]. Research into optoelectronic synaptic devices is primarily concerned with developing materials that can effectively store and process data using optical stimulation. Various materials, including oxide semiconductors, perovskites, graphene, and black phosphorus, are explored to enhance the efficiency and speed of these devices [16,17,18,19]. Particularly, oxide-based materials have traditionally been favored in neuromorphic systems due to their advantageous properties. Oxide materials, such as Fe_3_O_4_ nanodots and TiO_2_ nanowires, are known for their long-term stability, high current density, and reproducible switching characteristics, making them prominent choices for neuromorphic applications [10,20,21]. These materials offer a reliable performance and durability, which are crucial for mimicking biological synaptic functions. In contrast, carbon nanotubes (CNTs) are emerging as a promising alternative due to their unique properties, including high electrical conductivity, large surface area, and potential for high-density integration [22]. While CNT-based devices are less common than oxide-based ones, their ability to provide fast response times and versatile optoelectronic functionality make them an intriguing area of research [23].

In particular, the integration of nanostructured materials, such as carbon nanotubes (CNTs), has shown significant potential in improving optoelectronic efficiency and device response speed. CNT-based optoelectronic synaptic devices, known for their high density and large surface area, are particularly well-suited for high-density synapses in compact spaces. The extensive surface area of CNTs facilitates effective chemical interactions, crucial for processes like oxygen adsorption and desorption that influence device performance [24]. Despite their advantages, the performance of CNT-based devices can be affected by factors such as light reflection and noise, making material selection and design critical. Furthermore, optimizing CNT-based optoelectronic synaptic devices for neuromorphic computing requires the consideration of multiple factors beyond long-term memory, including material stability, synaptic weight modulation, response time, energy efficiency, synaptic density, and sensitivity to stimuli. While CNT-based devices have been predominantly explored in the field of photodetectors with a focus on achieving fast photoresponses, research on persistent photoconductivity (PPC) properties—crucial for neuromorphic computing applications—remains in its early stages [25]. This study aims to fabricate optically controllable CNT-based synaptic devices using CNT films applied to glass substrates and evaluate their impact on long-term memory characteristics. Specifically, we selected spin coating for fabricating CNT films due to its simplicity, scalability, and precise control over film thickness compared to other methods such as dip coating, spray coating, and chemical vapor deposition (CVD) [26,27,28]. Spin coating offers uniform coverage and reproducibility across large areas, which is crucial for consistent optoelectronic performance [27]. By varying the spin-coating speed, we can directly control the film thickness more precisely. This approach allows for more consistent and reproducible results, making it particularly suited for research applications where fine-tuning film properties is essential. While other techniques like CVD can produce high-quality films, spin coating’s cost-effectiveness and its ability to provide precise control over the film thickness make it ideal for investigating how CNT film thickness affects the device’s ability to mimic biological synaptic functions. This method allows us to explore the impact of CNT concentration on long-term memory retention, aiming to assess the effectiveness of optoelectronic synaptic devices in replicating the complex behaviors of biological synapses. 

## 2. Materials and Methods

A glass substrate was used to deposit the CNT films using the spin-coating process. The CNT solution was applied to the substrate and then processed using the spin-coating method. The CNT aqueous solution was provided by Cobon Inc., and ethanol was used as a solvent. For uniform application, the glass substrate was first cleaned organically. The substrate was ultrasonically washed for 10 min each in ethanol, isopropyl alcohol, and deionized water solutions. After organic cleaning, additional RCA cleaning was performed. RCA cleaning was carried out as follows: the substrate was immersed in a mixture of 200 mL deionized water and 40 mL of hydrogen peroxide, and 40 mL of ammonia at about 180 °C for 20 min. After cleaning the substrate, 10 drops of the CNT solution were dispensed onto the substrate. Subsequently, each sample was subjected to the spin-coating process for 30 s at varying RPM speeds from 2000 to 5000, thereby coating the substrate. The fabrication was completed by baking the substrate on a hot plate at 200 °C for 5 min. Finally, a 50 nm-thick Au electrode was deposited using a thermal evaporator. 

Various methods have been used to analyze the surface, crystallographic, optical, and electrical properties of CNT films. Atomic force microscopy (AFM) was used to measure surface structure, while Raman spectroscopy was used to analyze crystallographic properties. To evaluate the optical properties of CNT films, the optical transmittance was assessed using UV–visible spectroscopy. The dark current and UV current of a CNT-based optical synaptic device were evaluated by measuring the response to a 365 nm UV light source. To characterize the optical synaptic properties, the EPSC of the CNT-based optoelectronic synaptic device was measured at a voltage of 1.0 V. The optical potentiation process to induce EPSC using 365 nm UV stimulation involved varying parameters such as UV power, stimulation time, frequency, and the number of exposures. After UV termination, the EPSC value was gradually reduced through a natural depression process, where the EPSC value naturally decreased over time. Through this method, the learning and forgetting process was repeated twice. Based on the results, a visual memory simulation was conducted. In this study, the maximum EPSC value increased through learning was designated as 100%. During the forgetting process, visual memory images were generated by measuring the EPSC values at 50, 200, and 380 s intervals, respectively, and calculating the percentage of forgetting. The selected colors were smoothed in proportion to their reduced size. This method was applied to the second learning–forgetting cycle as well, and the images were implemented in a 3 × 3 array of cells. Overall, these analytical techniques have been used to understand the properties of CNT films as a function of spin coating RPM, which is important for optimizing the performance of optoelectronic synaptic devices.

## 3. Results and Discussion

**Figure 1:**
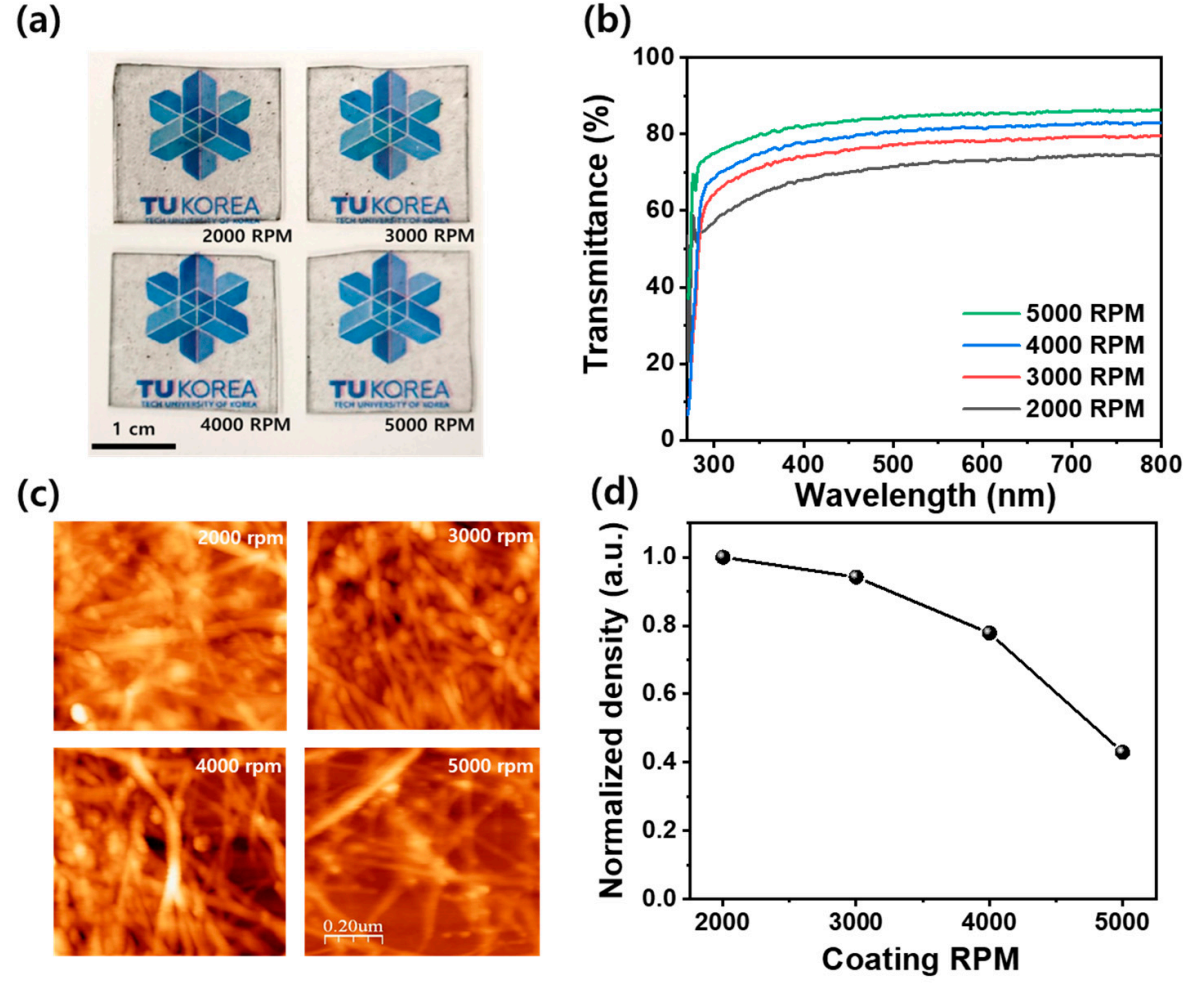
(**a**) Photographs of CNT/glass made by difference in spin-coating speed of CNT aqueous solution, (**b**) optical transmittance by CNT device manufactured according to spin-coating speed, (**c**) micro-surface shape images of CNT devices measured using an atomic force microscope, and (**d**) relative density graph of CNT devices.

Figure 1a shows CNT films with CNT aqueous solution applied on glass substrates using the spin-coating method. It indicates that the CNTs are uniformly coated all over the glass substrate, and, also, as the spin coating rotation speed increases, the transparent substrate becomes more transparent, allowing the image on the back side to be clearly seen. This indicates that, as the spin-coating speed increases, the CNTs are applied in a thinner layer, resulting in relatively high optical transmittance. The quantitative optical transmittance of the CNT film was measured by UV–visible spectroscopy, as depicted in Figure 1b. This is the optical transmittance of CNT films fabricated at spin-coating speeds from 2000 RPM to 5000 RPM, and the optical transmittance tends to increase over the entire region from 300 nm to 800 nm as the spin-coating speed of the CNTs increases. In particular, as the spin-coating speed increased from 2000 to 5000 RPM, the optical transmittance increased from 65% to 80% at 360 nm, and from 74.4% to 86.4% at 550 nm. This indicates that, at low spin-coating speeds, the optical transmittance decreases because the CNT coating forms a thicker film, scattering and absorbing more light. In particular, it can be seen that the optical transmittance of the CNT film is almost proportional to the spin-coating speed, which means that the thickness of the CNT film is well-controlled by the spin-coating speed [29]. Figure 1c shows the surface structures of the CNT film applied to the glass substrates using the different spin-coating speeds, measured using AFM. It is observed that the CNTs are well-applied by the spin-coating method regardless of the spin-coating speed, but it can be seen that the density of CNTs tends to decrease as the spin-coating speed increases. Figure 1d depicts the relative density of CNTs as a function of spin-coating speed, using the ImageJ program. It can be observed that the density diminished as the spin-coating speed increased from 2000 to 5000 RPM. These findings indicate that, as the spin-coating speed increases, the density of the CNTs declines, which reduces the scattering and absorption of light from the surface, thereby enhancing optical transmittance, as shown in Figure 1b.

**Figure 2:**
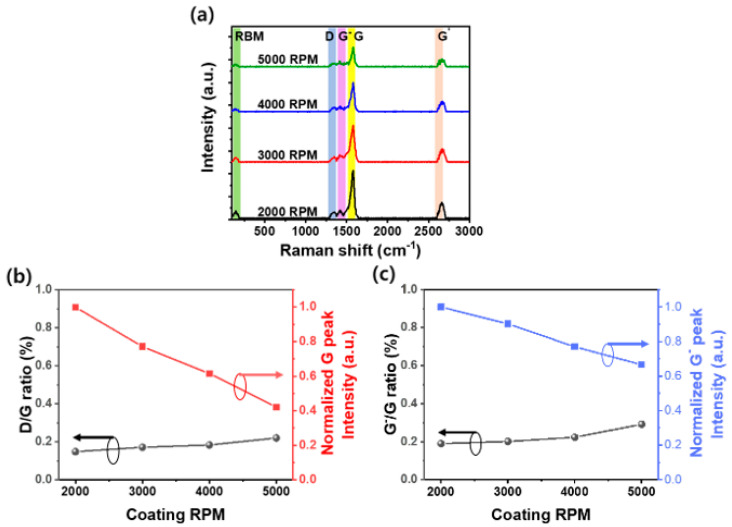
(**a**) Raman spectra of CNT film showing the RBM (green region), D (gray region), G^−^(pink region), G (yellow region), and G‘ (light peach color region) peaks under varying spin-coating speeds. (**b**) Normalized intensity of G and D/G peak ratio CNT film as a function of the spin-coating speed. (**c**) The area ratio of G^−^/G peaks and the normalized intensity of G^−^ peaks of CNT film as a function of the spin-coating speed.

Raman spectroscopy is a common method for analyzing CNT presence and structural defects without destroying the CNTs [30]. To analyze the characteristics of CNT films as a function of spin-coating speeds, the micro-Raman spectra of CNT films coated on the substrate at different spin-coating speeds are shown in Figure 2a. Regardless of the spin-coating speed, the five peaks associated with the radical breathing mode (RBM), D, G^-^, G, and G′, appear as clear peaks for 146, 1351, 1421, 1580, and 2661 cm^−1^, respectively [31]. In particular, the RBM of CNTs is a low-frequency mode that is well-known to have the strongest intensity in the Raman spectrum of CNTs, which is very useful for identifying and characterizing CNTs [32]. It can be observed that the peak intensity of the RBM decreases with increasing spin-coating speed. This is because higher spin-coating speeds can result in a thinner distribution of CNTs on the substrate. High frequencies in the D (defect), G (graphite), and RBM bands in the Raman spectrum can provide information about the defects in CNT films [33]. In general, the G-band, which exhibits a peak at around 1580 cm^−1^, arises from the in-plane vibrations of the hexagonal rings, common in all sp^2^-bonded carbon materials [34]. The ratio of the D/G bands in the Raman spectra of CNTs is often used to relatively evaluate the degree of defects in CNTs [35]. As shown in Figure 2b, the D/G ratio obtained by calculating the area of each spectrum through the fitting of the D and G peaks of each CNT film shows that the D/G ratio increases as the spin-coating speed increases. Moreover, it can be seen that the intensity of the relative D and G bands gradually increases as the spin-coating speed decreases. This suggests that more defects were formed in the thinly applied CNTs as the CNTs bumped into each other due to the increased speed in the spin-coating process, and the decrease in the intensity of the D and G bands with increasing spin-coating speed further suggests that the thickness of the CNT decreases. In detail, at higher spin-coating speeds, defects in CNT films primarily result from the rapid and uneven deposition of the material. The increased centrifugal force accelerates the spreading of the CNT dispersion, causing irregularities such as the aggregation and clustering of CNTs. This rapid spreading reduces the interaction time between CNTs and the substrate, leading to incomplete wetting and uneven film formation. The mechanical stress exerted by high-speed rotation also contributes to the deformation of CNTs, causing structural defects such as micro-cracks and voids. Additionally, the decreased wetting time prevents the solvent from evaporating uniformly, exacerbating the formation of surface irregularities. These defects, including the increased density of structural imperfections, are evident from the higher D/G ratio in Raman spectroscopy, reflecting the greater bending and deformation of CNTs [36]. Overall, these mechanisms result in a film with compromised electrical and photoelectric properties. Additionally, it is known that the G^−^ peak arises from the defects and disorder in the sp^2^ carbon lattice due to the curvature of the CNT. The presence and intensity of the G^−^ peak provide information about the amount of structural disorder or number of defects within the CNTs. However, the G^−^ peak is related to the splitting of the G band due to the curvature of the CNT and is less associated with defects compared to the D peak [37]. As shown in Figure 2c, when the spin-coating speed increases from 2000 to 5000 RPM, the relative intensity of the G^-^ peak decreases by approximately 40% compared to the intensity of CNT film coated at 2000 RPM. This decrease suggests that a higher spin-coating speed reduces the curvature of the CNTs due to centrifugal force, leading to fewer defects and less disorder. However, as the spin-coating speed increases from 2000 RPM to 5000 RPM, the G^-^/G ratio still increases. This is because, when the spin-coating speed reaches 5000 RPM, the CNT film becomes thinner, as shown in Figure 1, resulting in a 60% reduction in the G peak of the CNT film coated at 5000 RPM compared to the CNT film coated at 2000 RPM, which is greater than the 40% decrease in G^-^ peak intensity. Based on these results, it is believed that CNT thin films coated at high spin-coating speeds have fewer defects related to bending due to the reduced curvature of the CNTs. However, at a high spin-coating speed, the increased collision between CNTs can lead to the formation of larger defects.

**Figure 3:**
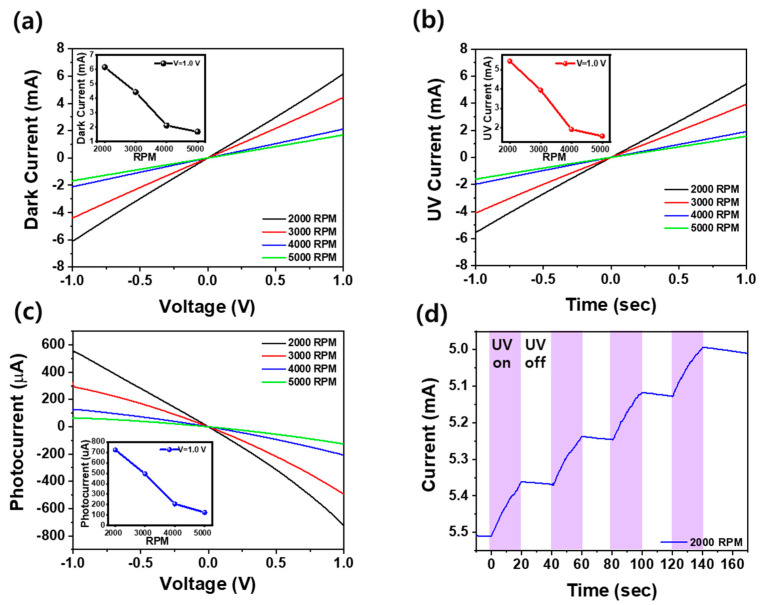
(**a**) Dark current, (**b**) UV current, and (**c**) photocurrent of Au/CNT/Au devices with spin-coating speeds ranging from 2000 to 5000 RPM. The inserts in (**a**−**c**) show the dark current, UV current, and photocurrent measured at 1.0 V as functions of the spin-coating speed of the CNT film, respectively. (**d**) The variation in photocurrent over time for an Au/CNT/Au device coated at 2000 RPM, which is subjected to 365 nm UV light for 20 s, followed by 20 s of no irradiation, with the cycle repeated five times.

Figure 3a shows the dark current (I_dark_) of the CNT films as a function of applied voltage when the spin-coating speed was increased from 2000 RPM to 5000 RPM. Regardless of the spin-coating speed, the CNT films exhibited a high operational current of more than 1.0 mA at 1.0 V. This suggests that the CNT films deposited by the spin-coating method are physically well-connected, resulting in relatively high operating current. In addition, as the spin-coating speed increased from 2000 to 5000 RPM, the dark current decreased from 6.16 mA to 1.69 mA at 1.0 V, respectively. This decrease in dark current is likely due to the reduced thickness of the CNT film at higher spin-coating speeds, which results in a decrease in the current path, and, thus, lower conductivity, leading to a decrease in the operating current. Figure 3b shows a graph of the UV current (I_uv_) versus applied voltage for CNT films exposed to UV light, with spin-coating speeds ranging from 2000 to 5000 RPM. The I–V characteristics of the CNT films after UV exposure were similar to those of the dark current; however, all CNT films exhibited an increase in resistance, resulting in a decrease in operating current. In particular, the dark current of the CNT film applied at 2000 RPM was 6.16 mA at 1.0 V before UV exposure, as shown in Figure 3a, but the UV current decreased to 5.44 mA after UV application. Furthermore, all CNT films showed UV current values lower than the dark current, regardless of the spin-coating speed.

Figure 3b shows a graph of the UV current (I_uv_) versus applied voltage measured while exposing the applied CNT film to UV light while increasing the spin-coating speed from 2000 to 5000 RPM. The I–V curves of the CNT films after UV exposure were observed to be similar to the dark current, but all CNT films showed an increase in resistance, resulting in a decrease in operating current. In particular, the dark current of CNT film applied at 2000 RPM was found to be 6.16 mA at 1.0 V before UV exposure, but the UV current was found to decrease to 5.44 mA after UV application. Furthermore, all samples showed UV current values lower than the dark current regardless of spin-coating speed. Based on these results, to determine the photocurrent of CNT films, a negative photocurrent can be observed by subtracting the dark current from the UV current, as shown in Figure 3c [38]. This characteristic is due to the p-type nature of the CNT film. When UV light desorbs oxygen molecules adsorbed on the surface of the CNT film, the trapped electrons are released and enter the CNTs. These electrons recombined with the holes, which are the majority carriers of p-type CNTs. This recombination reduces the number of free holes, increasing the resistance and resulting in a negative photocurrent [39]. In particular, as the spin-coating speed of CNT decreases, the negative slope of the photocurrent of CNT increases. This means that, as the spin-coating speed decreases, the CNT film becomes thicker and denser, leading to increased resistance due to more O_2_ desorption from the larger surface area of the thicker CNTs [40]. The inset of Figure 3c shows a graph of the photocurrent obtained at 1.0 V after UV exposure to a CNT film device as a function of spin-coating speed of the CNT film. It shows that the photocurrent tends to decrease almost linearly from 724 μA to 126 μA as the spin-coating speed increases from 2000 RPM to 5000 RPM. This indicates that the thickness of the CNT film can be effectively controlled by adjusting the spin-coating speed, and, thus, the magnitude of the photocurrent can be precisely regulated. Figure 3d shows the time-dependent photocurrent of a device annealed at 2000 RPM, measured over four cycles of 20 s of UV exposure followed by 20 s with the UV light off. A relatively fast negative photocurrent is observed during UV exposure, followed by a very slow recovery after the light is turned off. This behavior is attributed to the rapid desorption of oxygen during UV exposure and the slow reabsorption afterward, suggesting the potential for long-term optical memory devices and the suitability of CNT-based devices for LTM applications.

**Figure 4:**
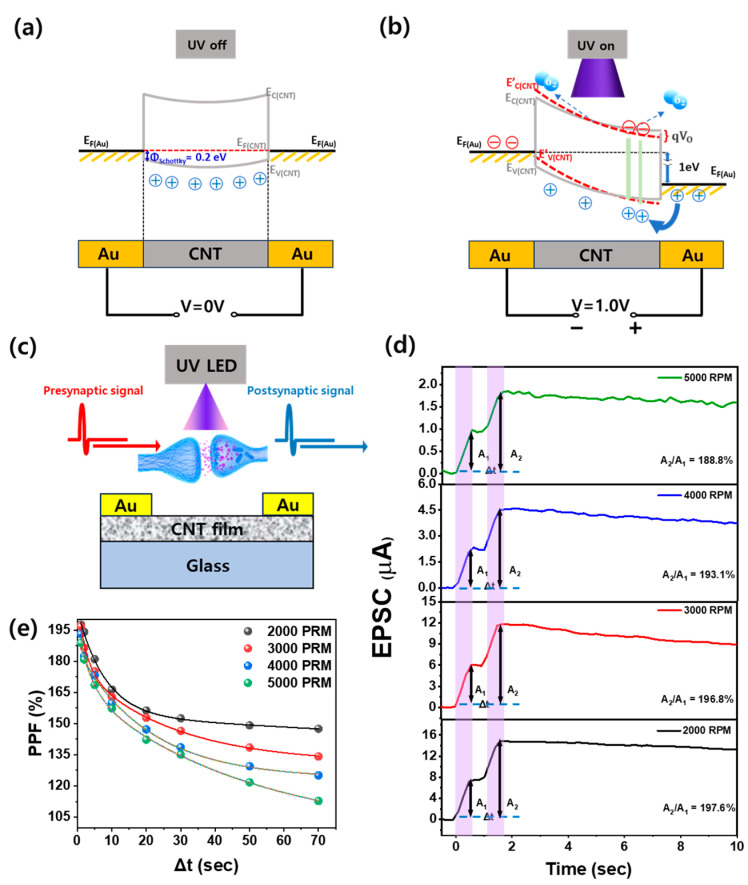
Energy band diagram of an Au/CNT/Au optoelectronic synaptic device under (**a**) dark state with an applied voltage of 0 V and (**b**) UV-exposed state with an applied voltage of 1.0 V. (**c**) Schematic representation of an Au/CNT/Au optoelectronic synaptic device fabricated on glass substrate, illustrating the transmission of presynaptic and postsynaptic signals at the synapse upon UV application. The CNT-based optoelectronic synaptic device was coated at 2000 to 5000 RPM, (**d**) time-dependent variation of EPSC at 1.0 V when UV was applied in two consecutive light pulses with 0.5 s exposure and 0.5 s off time, and (**e**) PPF variation with increasing light off time (Δt) with a constant 0.5 s pulse exposure time.

The work functions of Au and CNT are 5.1 eV and 4.9 eV, respectively [41,42]. When a metal–semiconductor interface forms, the band bending is determined by the Schottky barrier height of 0.2 eV, expressed as qΦ_Bi_ = q(Φ_Au_ − Φ_CNT_) [43]. Therefore, at equilibrium with no external voltage applied, a Schottky barrier of approximately 0.2 eV forms at the Au–CNT interfaces due to this band bending. Since identical Au electrodes are used on both sides of the CNT, a symmetrical band profile is observed, as shown in the Figure 4a. When a positive voltage of 1.0 V is applied to the Au electrode to measure the UV-induced photocurrent, the bands bend downward by an amount corresponding to qV, leading to an energy shift of 1.0 eV, as shown by the gray solid line in Figure 4b. Upon UV exposure, oxygen ions adsorbed on the surface of the CNT film are desorbed, releasing oxygen atoms and introducing excess electrons into the CNT. This increases the potential energy, shifting the band upward, as indicated by the red dotted line in Figure 4b. The desorbed oxygen atoms release electrons, which recombine with holes, the majority carriers in the p-type CNT, reducing the hole concentration and thereby increasing the resistance of the CNT [39]. This resistance change gives rise to the observed photocurrent. After the UV stimulation ends, oxygen molecules are re-adsorbed onto the CNT surface, trapping electrons and reducing the potential energy. This causes the band to bend downward again, decreasing resistance and improving conductivity [44]. Figure 4c shows a schematic of an Au/CNT/Au optoelectronic synaptic device and the transmission of presynaptic and postsynaptic signals at the synapse. Following this, Figure 4d shows a plot of EPSCs over time for an Au/CNT/Au synaptic device exposed to UV light for 0.5 s followed by the termination of UV exposure for 0.5 s, repeated twice. The EPSC induced by UV exposure tended to decrease as the spin-coating speed of CNTs was increased from 2000 to 5000 RPM. This trend is similar to the behavior observed in the photocurrent as shown in Figure 3c. In addition, the rapid production of EPSCs by UV stimulation in all devices and the slow decrease in EPSCs after the end of UV stimulation were observed. This characteristic is because UV stimulation causes oxygen desorption in CNTs with p-type properties, while O_2_ molecules are re-adsorbed on the CNT surface after UV stimulation ends. The desorption process of oxygen molecules due to UV light stimulation occurs quickly, resulting in a rapid increase in resistance. Conversely, the adsorption process of oxygen molecules after UV light termination occurs slowly, inducing a gradual recovery to the baseline. In particular, at 5000 RPM, the EPSC showed a relatively small increase and a relatively sharp decrease after the end of the UV stimulation. However, at 2000 RPM, it showed a higher EPSC upon UV stimulation and maintained a higher EPSC for a longer period after the UV stimulation was terminated. This behavior is due to the desorption of many oxygen molecules by UV stimulation on the larger CNT surface area in the device where CNTs are more densely packed, followed by the slow adsorption on the large CNT surface area after UV stimulation termination. This slow decrease in EPSC is an important indicator that devices with a high CNT density exhibit better synaptic activation levels and memory properties. It indicates that CNT optical synaptic devices fabricated with low spin-coating speeds have better short-term plasticity. In addition, the EPSC value increased by the first UV stimulation is called A_1_, and the EPSC value increased by the second UV stimulation is called A_2_. The A_2_/A_1_ values, that is, PPF, which is the ratio of the EPSC to the postsynaptic potential, were compared by spin-coating speed. It can be seen that, as the spin-coating speed decreases, the A_1_ and A_2_ values for the primary and secondary UV exposure increase.

Additionally, all devices, regardless of spin-coating speed, maintained high EPSCs when the UV light was turned off after the secondary UV exposure. This indicates that the CNT-based synaptic devices exhibit PPC characteristics, where the increased resistance upon UV excitation does not recover quickly and remains in a state of high photocurrent. In addition, in biological and optoelectronic systems, a larger initial EPSC (A_1_) can reduce the magnitude of facilitation (A_2_/A_1_) in the PPF due to a greater release of neurotransmitters (or charge carriers in the case of devices) during the first pulse, leaving less available for the second pulse. This would typically result in lower PPF values for devices with higher initial EPSC values. However, devices fabricated at a lower coating speed (2000 RPM) not only exhibit higher EPSC values but also demonstrate an increase in PPF. This result suggests that CNT-based synaptic devices fabricated at 2000 RPM possess superior immediate synaptic responses and short-term plasticity properties simultaneously. Figure 4e shows the PPF value as the time (Δt) between UV stimulation intervals of CNT synaptic devices increases according to the speed of spin coatings. As Δt increased from 0.5 s to 70 s in the 5000 RPM device, the PPF index decreased rapidly from 188.8% to 113%, confirming that the short-term memory characteristics rapidly disappeared. This indicates that the gradual decrease in PPF value with an increasing stimulation exposure interval is consistent with the time dependence of the characteristics of optical synaptic devices and the PPF index of biological synapses, where shorter intervals between stimuli result in a higher PPF index [45]. However, as Δt increased from 0.5 s to 70 s in the 2000 RPM device, the PPF index decreased more slowly, from 198.5% to 147.5%, indicating that the long-term memory characteristics can be maintained for a longer time. This trend is further highlighted by the difference in PPF between the CNT coating at 2000 RPM and those coated at 5000 RPM, becoming more pronounced as Δt increases, with values of 147.5% and 112.8%, respectively, at Δt of 70 s. Since PPF is an important indicator of the long-term memory properties of synapses, the increase in PPF with decreasing spin-coating speed suggests that the long-term memory properties of the optoelectronic synaptic devices are enhanced. This enhancement is due to the formation of thicker and denser CNT films at lower spin-coating speeds, which increases the number of available charge-trapping sites. These trapping sites help sustain the photogenerated carrier for longer periods, thereby enhancing the PPC effect.

**Figure 5:**
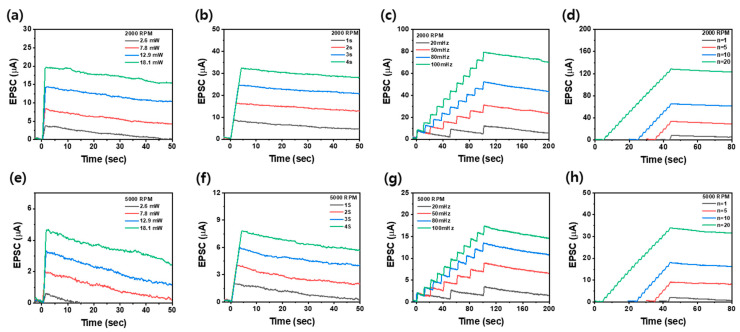
EPSCs as a function of time for Au/CNT/Au optoelectronic synaptic devices coated using spin-coating speeds of (**a**–**d**) 2000 RPM and (**e**–**h**) 5000 RPM, with varying (**a**,**e**) UV power, (**b**,**f**) exposure time, (**c**,**g**) exposure frequency, and (**d**,**h**) exposure numbers.

Figure 5a–d and Figure 5e–h show the EPSCs obtained under different UV stimulation conditions for CNT-based optoelectronic synaptic devices coated at spin-coating speeds of 2000 and 5000 RPM, respectively, and the EPSC decay with time after the end of UV exposure. The optical potentiation of a synaptic device through UV stimulation was controlled by the UV exposure power (2.6 mW–18 mW), duration (1–4 s), frequency (20–100 mHz), and number of exposures (1–20). Regardless of the spin-coating speed, it is found that all synaptic devices responded immediately with an increase in EPSC at the beginning of stimulation under different UV exposure conditions. Additionally, the EPSC did not decrease rapidly after the end of UV stimulation but remained for a long time, attributed to the PPC effect. This indicates that the optical potentiation is well-formed, and the long-term memory properties are maintained by UV stimulation even in thin–thickness CNT synaptic devices. Notably, CNT-based synaptic devices fabricated at the lower spin-coating speed of 2000 RPM exhibited higher EPSCs under the same UV stimulation conditions compared to those coated at 5000 RPM. In particular, depending on factors such as UV light output, exposure time, frequency, and number of exposures, thick CNT synaptic devices coated at 2000 RPM showed an increase in EPSC values from 3.7 μA to 130 μA, while thin CNT synaptic devices coated at 5000 RPM showed an increase in EPSC values from 0.6 μA to 34 μA. This result is due to the decreased spin-coating speed, which results in more CNTs being deposited, thereby increasing the CNT density. This increased density facilitates the desorption of a large amount of oxygen molecules adsorbed on the surface, leading to the formation of higher EPSCs. Furthermore, CNT-based optoelectronic synaptic devices with a higher CNT density and those exposed to different UV stimulation conditions maintained EPSCs for a longer period of time after the UV stimulation ended. Thicker CNT devices and those subjected to higher UV stimulation exhibit a delayed natural depression due to the difference in the desorption and adsorption rates of oxygen molecules on the CNT surface. During UV stimulation, the oxygen molecules desorb rapidly from the CNT surface because the UV light weakens the bond between the oxygen molecules and the CNT surface. After the UV stimulation ends, these oxygen molecules reabsorb on the CNT surface more slowly. The rapid desorption occurs because the UV light provides the activation energy needed for oxygen molecules to detach from the surface. In contrast, reabsorption happens slowly as the oxygen molecules interact with electrons within the CNTs due to electron affinity. This slow reabsorption process allows the EPSC to be maintained for a longer time [46]. Consequently, thicker CNT-based optoelectronic synaptic devices with a higher density, and those exposed to higher UV stimulation, sustain their EPSCs longer, thereby exhibiting enhanced long-term memory characteristics [11].

**Figure 6:**
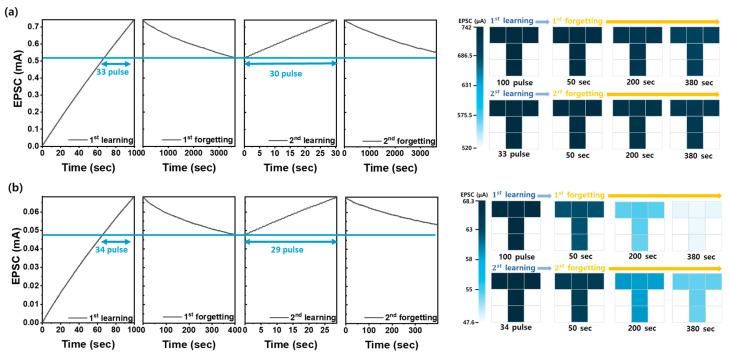
The measured learning and forgetting experience behaviors in response to optical stimuli for CNT-based optoelectronic synaptic devices manufactured at (**a**) 2000 RPM and (**b**) 5000 RPM. A pulsed optical stimulus (365 nm UV light with a pulse width of 0.5 s and a 50% duty cycle) was applied 100 times to induce first learning, followed by two cycles of forgetting after turning off the UV light. To form human visual memory, nine devices were selected to visually represent the forgetting process after the first and the second learning processes, aiming to mimic human visual memory.

To investigate the learning and forgetting processes of CNT-based optoelectronic synaptic devices, the optical learning process was measured by observing a gradual increase in EPSCs in response to UV light pulses, which leads to an increase in synaptic weight. The forgetting process was examined through natural depression, characterized by a gradual decrease in EPSCs after the UV light was turned off [12]. Figure 6a,b illustrate the learning and forgetting processes of CNT-based optoelectronic synaptic devices fabricated at spin-coating speeds of 2000 RPM and 5000 RPM, respectively. The learning process was induced by UV light pulses (1.0 s period, 50% duty cycle) repeated for 100 cycles, during which the EPSCs were monitored to observe the optical potentiation. Following the 100 cycles of UV light stimulation, the forgetting process was examined by observing the natural depression of EPSCs. This sequence of learning and forgetting was repeated twice to determine the number of pulse cycles required for learning and the time required for forgetting at each stage. In the learning process, the EPSC obtained after 100 repetitions of optical potentiation was defined as the maximum EPSC, with the learning process considered successful when the EPSC reached more than 70% of this maximum EPSC. During the forgetting process, the EPSC was considered forgotten when the EPSC fell below 70% of the maximum EPSC during natural depression following the cessation of UV light stimulation. During the first learning process, each device achieved the maximum EPSC using 100 UV pulse stimulations. For the CNT spin-coating speed at 2000 RPM, the EPSC increased from 0 to 742.2 μA, while, at 5000 RPM, it increased from 0 to 68.27 μA. Despite the maximum EPSC decreasing with a higher spin-coating speed, both devices reached their maximum EPSCs after 33–34 pulse injections. Notably, the CNT optoelectronic synaptic device coated at 2000 RPM maintained an EPSC of more than 70% for 3600 s after the UV light was turned off during the first forgetting process. This duration is about 9 times longer than the learning retention time (400 s) of the CNT optoelectronic synaptic device coated at 5000 RPM. This result indicates that the long-term memory properties were significantly improved as the spin-coating speed of the CNTs decreased. Furthermore, a decrease in the number of pulses required to induce the maximum EPSC was observed during the second learning process in successive repetitions of pulse injections. For CNT optoelectronic synaptic devices coated at spin-coating speeds of 2000 and 5000 RPM, the number of pulses required to reach the maximum EPSC range from 33 to 30 and from 34 to 29, respectively. This indicates that the second learning process reaches the maximum EPSC value faster through repeated optical pulse injections compared to the first learning process. Particularly noteworthy is that the CNT optoelectronic synaptic device coated at 2000 RPM showed EPSCs remaining above 70% for the entire 3600 s during the two UV termination cycles. Moreover, to quantify the rate of forgetting, Wickelgren’s power law was employed. This law is a well-established model for describing biological forgetting and is represented by the following equation (Equation (1)) [10,47]:

I = λ × (1 + β × t)^−Ψ^ (1)

where I is the memory strength, t is the decay time, λ is the state of long-term memory at t = 0 (degree of learning), β is the scale parameter, and Ψ is the forgetting rate. The learning degrees for the first forgetting process were calculated to be 4.69 × 10^−6^ for devices coated at 5000 RPM and 1.98 × 10^−5^ for those coated at 2000 RPM, indicating a significant improvement in long-term memory properties with an increased CNT film thickness. The forgetting rate was notably reduced from 2156 to 0.38587, reflecting enhanced retention capabilities. Although this forgetting rate is higher than the value of 0.138 reported for Ga_2_O_3_-based optoelectronic synapse devices [10], it underscores the remarkable performance achieved with simple spin-coated CNT films. Thus, it is confirmed that the spin-coating speed of CNTs has a positive effect on the long-term memory properties of CNT-based optoelectronic synaptic devices, with a higher CNT density achieved at lower spin-coating speeds. Additionally, a 3 × 3 array was operated to confirm the uniformity of the CNT optoelectronic synaptic device, and visual simulation experiments were performed to observe the behavior of EPSCs in this array. Nine adjacent CNT optoelectronic synaptic devices from a single wafer were selected to represent the 3 × 3-pixel layout. Each device was stimulated for 100 s, comprising 100 pulses with a pulse width of 0.5 s and a duty cycle of 50%. The EPSC values for these pulse inputs were used to encode the predefined pixel colors and intensity into the devices. A 3 × 3 array was used to observe the decay of EPSCs over time after achieving maximum EPSCs in the first and second learning–forgetting processes. The CNT optoelectronic synaptic device coated at 5000 RPM showed rapid memory decay, with EPSCs nearly extinguished within 380 s in the first forgetting process. However, in the second forgetting process, the device showed a slower decay of the EPSC, indicating it remained in a higher learning state. In contrast, the CNT optoelectronic synaptic device coated at 2000 RPM retained a color similar to the state within 380 s in both the first and second forgetting processes. When comparing the color representing the relative learning state 380 s after UV termination, the CNT optoelectronic synaptic device coated at 2000 RPM exhibited a dark blue color, while the CNT optoelectronic synaptic device coated at 5000 RPM appeared much lighter. This indicates that the CNT optoelectronic synaptic device coated at 2000 RPM maintains better long-term memory properties than the one coated at 5000 RPM. This suggests that the CNT optoelectronic synaptic device fabricated at lower spin-coating speeds, which result in a higher CNT density, can better sustain long-term memory due to the enhanced interaction with oxygen in the CNT film, leading to more persistent memory characteristics.

## 4. Conclusions

The spin-coating speed during the fabrication of CNT-based optoelectronic synaptic devices plays a crucial role in determining their long-term memory properties. Devices coated at lower spin speeds (2000 RPM) exhibit thicker and denser CNT films, which lead to higher EPSCs and enhanced PPC due to the increased number of charge-trapping sites. This enhanced PPC effect allows these devices to maintain higher EPSCs for extended periods after UV stimulation has ended, thereby demonstrating superior long-term memory retention compared to devices coated at higher spin speeds (5000 RPM). The results show that devices with denser CNT films can sustain memory states longer, with a slower EPSC decay, making them more effective in mimicking biological synaptic behavior. Furthermore, the reduced number of pulses required to achieve the maximum EPSC in successive learning cycles indicates improved learning efficiency with repeated training. These findings suggest that, by optimizing the spin-coating speed, the performance of CNT-based optoelectronic synaptic devices can be significantly enhanced, making them promising candidates for applications in neuromorphic computing and artificial neural networks, where long-term memory retention and efficient learning are critical.

## Data Availability

The data presented in this study are available on request form the corresponding author.
